# Supplementary material for: Are carbon emissions trading and green financial instruments synergistic? -Comprehensive quantitative research based on content analysis
Source: PLoS One. 2024 Mar 7;19(3):e0298601. doi: 10.1371/journal.pone.0298601 (PMC10919601; doi:10.1371/journal.pone.0298601)
Supplement: S1 Data — (ZIP) [file pone.0298601.s001.zip › Data/地级市Z_F工作报告.zip!/╔╜╢1⁄2/╟α╡║╩╨/└┤╫╘╣1⁄2╓┌║┼í╛┬φ┐╦╩2╛▌═°í┐_2013.docx]

各位代表：

　　现在，我代表市人民政府向大会报告工作，请予审议，并请各位政协委员和其他列席人员提出意见。

　　一、2012年工作回顾

　　2012年是本届政府工作的第一年，也是落实市第十一次党代会精神的第一年。在中共青岛市委的领导下，我们认真贯彻科学发展观，把握稳中求进的工作总基调，落实市第十一次党代会提出的目标任务，努力以世界眼光谋划未来，以国际标准提升工作，以本土优势彰显特色，面对复杂的经济形势和艰巨的发展任务，解放思想，改革创新，攻坚克难，奋力突破，展开和实施新一轮发展布局，在率先科学发展、实现蓝色跨越，加快建设宜居幸福的现代化国际城市道路上迈出坚实步伐。

　　预计，全市生产总值增长10.5%左右，公共财政预算收入增长18.4%，固定资产投资增长22%以上，社会消费品零售总额增长14.5%左右，城市居民人均可支配收入、农民人均纯收入分别增长12%左右、14%左右，城镇登记失业率控制在3%以内，居民消费价格上涨2.7%，人口自然增长率2.37‰，完成了节能减排各项任务。

　　一年来，主要做了以下工作：

　　（一）实施全域统筹发展战略

　　围绕建设宜居幸福的现代化国际城市目标，制定了指标体系和工作计划，完成了城市总体规划修编和新一轮行政区划调整，高起点推进全域规划建设。

　　推进城乡一体化建设。统筹资源、要素向薄弱区域倾斜，出台了重点支持两区两市发展的意见，制定了统筹城乡发展、提升县域整体实力、推进小城镇和新型农村社区建设的政策措施，开展了新一轮镇域产业发展和社区建设规划。开工建设新型农村社区102个，新建农村住房2.7万套，改造农村危旧房4067户。启动主城区危旧房改造22个片区。城镇化率达到69%左右。

　　推进三城联动、轴带展开。黄岛、红岛和老市区功能互补、联动发展，基础设施轴带分布、加快建设。青龙高速、地铁2号线、重庆路改造开工建设，快速路三期、海底隧道和海湾大桥接线基本完成，铁路青岛北客站、青荣和海青铁路项目进度加快。完成了蓝色硅谷城际铁路规划，邮轮母港一期工程开工建设。市容环境整治大干300天行动取得新成效，城市环境进一步改善。

　　推进生态间隔、组团发展。大沽河综合治理全线开工，17公里标准段堤防基本建成。世园会主展馆封顶，主园区基础设施工程完工。在植树增绿行动中，新改建绿地731公顷，开工建设11处万亩林场，新增造林面积14万亩。编制完成环胶州湾保护控制线划定与岸线整理规划,海泊河等入湾河流整治工程加快。可再生能源建筑应用新增300万平方米。加强大气、污水专项治理，淘汰高污染黄标车2.4万辆，开展了细颗粒物监测。加快董家口港城、新河新城、姜山新城、交通商务区等组团的开发建设，新规划各层级发展片区21个，规划面积达2200平方公里，组团发展格局正在形成。

　　（二）加快蓝色经济区建设

　　大力培育发展蓝色产业，制定了蓝色跨越三年行动计划。140个蓝色重点产业项目开工率达到71%。海洋经济增加值突破1100亿元，增长20%左右。加快“一谷两区”规划建设。西海岸经济新区规划编制完成，重点片区开发已启动，加快打造现代化国际新城区。中德生态园、中日韩创新产业园加快推进，青岛港30万吨级矿石码头建成运营，港口和集装箱吞吐量分别达到4.1亿吨、1450万标准箱。董家口经济区已入驻项目计划总投资3000亿元。蓝色硅谷核心区加快启动。山东大学青岛校区、青岛海洋科学与技术国家实验室、国家深海基地等创新平台建设加快推进。红岛经济区新引进清华科技园等一批投资过10亿元的重大项目，软件科技城加快建设。

　　（三）加快创新转型步伐

　　推进创新体系建设。千万平米科技孵化器建设已开工230万平方米，竣工118万平方米。千万平米软件产业园初步形成“东园西谷北城”发展格局，软件业收入增长35%。成为首批国家级软件和信息服务业示范基地。千万平米人才公寓建设已开工128万平方米，竣工56.6万平方米。新一轮人才行动计划当年引进的硕士、博士等高层次人才增长了53%。全市发明专利申请量、授权量分别增长84%和35%。引进中科院声学所分所、中国家用电器研究院等一批国内外高端研发机构，支持发展企业创新主体，组建了11家产业技术创新战略联盟。中船重工710所、中海油重质油加工研发基地等一批大企业科研院所落户，海信研发基地等项目开工。省属大企业研发集群基地已启动规划。

　　促进创新转型发展。十条工业千亿级产业链产值增长15%。新启动老城区12户工业企业搬迁，青钢迁建获批。县域先进制造业园区规模扩大，规模以上工业产值增长24%。战略性新兴产业产值增长18%，占规模以上工业比重达到23%，高新技术产业产值占规模以上工业比重达到39.9%。高端服务业十个千万平米工程已开工在建2800万平方米，竣工1000万平方米。引进金融机构17家，市农商行、海协信托重组开业。新增贷款余额1130亿元，金融业增加值增长17%。新增上市企业3家。物流、旅游、商贸、中介服务也有新发展。现代农业加快发展，发放农业四项补贴6.9亿元，实施了“双百双千”工程，完成四个百万亩示范区建设，粮食总产再创历史新高。对42座病险水库进行改造，完成30个高效节水灌溉工程。渔业标准化建设有新进展。

　　（四）深入推进改革开放

　　推进了市属竞争性国有企业改革，整合国有平台公司资源，规范国有市政类企业，组建国有文化公司，推进国有各类企业现代企业制度建设。理顺重点发展区域管理体制，强化和完善经济发展功能。调整了财政体制，增强区市财政保障能力。强化小微企业扶持政策和支持民间投资措施，非公有制经济增加值增长10%左右。

　　完善招商引资和投资促进体制。对接央企签约项目28个，引进世界500强项目14个，到账外资46亿美元，增长26.6%。对出口企业给予扶持，外贸进出口增长4.5%，离岸服务外包执行额增长1.8倍，新批境外投资、对外承包工程营业额分别增长1.4倍和1.3倍，并购国外品牌企业取得突破。胶州经济开发区升级为国家级开发区，青岛保税港区汽车整车进口口岸获批。与巴塞罗那等三个城市缔结国际友城关系，与日本京都、韩国釜山成为经济合作伙伴城市，开通了青岛至法兰克福和洛杉矶国际航线。与烟台等周边五市签署了全面战略合作框架协议，积极完成对口支援工作任务，区域合作得到加强。

　　（五）全力保障和改善民生

　　市财政对民生投入增长21.6%，占财政支出比重达到60.8%，为城乡居民办的10件实事和6件新增民生事项如期完成。全市新增就业41.7万人。区、市最低工资标准分别提高12.7%和15.8%，城乡居民养老保险基础养老金翻了一番，企业退休人员养老金提高14.5%，城乡低保标准分别提高14.3%和15.2%，农村扶贫标准提高了两倍。新增养老机构床位3200张。开工保障性住房建设项目31个，筹建房源2.4万套，配租、配售房源9214套。市区集中供热普及率达到73%。新增大型商贸设施60万平方米，完成15处农贸市场标准化升级改造，建成肉菜流通追溯体系。

　　启动了千万平米社会事业公共设施建设，已开工272万平方米。坚持教育优先发展，预算内教育经费支出占财政支出比重提高到17%。启动百万平米现代化教育设施建设，完成94所农村薄弱中小学改造，新建、改扩建253所农村幼儿园。免除农村中职学生学费和全市义务教育段学生教科书、作业本费用。配备623辆专用校车。加快公立医院改革，基本和重大公共卫生服务实现城乡全覆盖，全市政府办基层医疗卫生机构和规划内的村卫生室全部实施基本药物制度，建立了城乡重大疾病、罕见疾病医疗救助制度，城镇居民医保和新农合政府补助标准提高到每人每年240元以上。完善基层公共文化服务体系，市博物馆改扩建等项目加快推进。活跃群众性体育健身活动，新增社区健身设施778处，青岛籍奥运选手在伦敦奥运会获得两金一银的好成绩。国防教育和民兵预备役工作取得新成绩，圆满完成中俄联合军演保障任务，我市第七次被命名为全国双拥模范城。人口计生、妇女儿童、慈善、残疾人、老龄事业进一步发展，外事、侨务、对台、统计、档案、史志、仲裁、人防、气象、民族宗教、防震减灾等工作不断加强。

　　加强社会治安防控，依法严厉打击刑事犯罪，努力建设平安青岛。健全公共安全应急体系。着力加强社区管理与服务。基层社会管理创新综合试点取得成效。开展“问安青岛”活动，建立了安全月、安全周制度，强化道路交通、消防等重点领域的安全监管，事故总起数下降8.9%。开展“食安青岛”整治行动，查办食品违法案件4930起。推进领导干部大接访、带案下访工作，信访积案和矛盾纠纷得到有效化解。圆满完成了十八大安保维稳任务。

　　（六）切实加强政府自身建设

　　坚持依法行政。自觉接受人大、政协和社会监督，向市人大常委会提请审议4件地方性法规草案，制定市政府规章10件。办复人大代表议案、建议和政协提案、建议案1516件。深入开展行政程序年活动。推进各级财政预决算公开，加大政务公开力度。推行电子政务，畅通网络在线问政、行风在线等政民互动渠道，完善便民服务体系。

　　努力优化经济发展环境。行政审批效率提速32%，市级行政审批事项减少19%。建立政府联系服务企业制度，暂停11项涉企行政事业性收费和降低16项经营服务性收费标准，为企业减轻税费负担8亿元。对损害发展环境行为进行问责，行政处罚权规范透明运行，效能监察力度加大。提出加速提升创新增效落实的要求，修订完善政府工作规则，治理庸懒散慢拖瞒行为，营造干事创业氛围。健全制度与惩治腐败结合，政府廉政建设进一步加强。

　　各位代表！成绩的取得，是党中央国务院、省委省政府和市委正确领导的结果，是市人大、市政协和社会各界有效监督、大力支持的结果，是全市上下同心协力、拼搏进取的结果。在此，我代表市人民政府，向全市人民，向人大代表和政协委员，向中央、省驻青单位，向驻青部队和武警官兵，向社会各界人士和离退休老同志，向所有关心支持青岛发展的海内外朋友们，表示衷心的感谢和崇高的敬意!

　　当前，国际经济、政治形势依然错综复杂，我市经济社会发展面临不少困难和挑战，政府工作也依然存在差距和不足。外需持续低迷，稳出口压力大；创新能力不强，转方式调结构困难多；城乡和区域发展不协调问题依然突出；许多方面的改革突破不大，率先科学发展的体制机制还要强化；一些重点项目、重点区域的建设进度不快，抓推进、抓落实的力度不够；许多群众生活困难，居民收入、就业、住房、教育、医疗、保障等民生问题需进一步解决好；政府工作的民主公开、依法行政、服务效率方面存在的问题亟待改进，消极腐败现象仍有发生，工作作风转变不够。这些问题都必须加以解决。

　　二、2013年主要工作安排

　　今年是全面贯彻落实党的十八大精神的开局之年。做好政府工作，必须深刻领会十八大的主题，深刻领会科学发展观的历史地位和指导意义，深刻领会建设中国特色社会主义的总依据、总布局和总任务，深刻领会全面建成小康社会和全面深化改革开放的目标，深刻领会社会主义经济建设、政治建设、文化建设、社会建设、生态文明建设等方面的重大部署，牢牢把握大有可为的重要战略机遇期，不断增强忧患意识、创新意识、宗旨意识、使命意识，振奋精神，拼搏进取，努力使各项工作走在全国全省前列，为力争到2016年实现全市生产总值和城乡居民人均收入比2010年翻一番，率先全面建成更高水平的小康社会打好坚实基础。

　　今年政府工作的总体要求是：坚持以邓小平理论、“三个代表”重要思想、科学发展观为指导，全面贯彻落实党的十八大精神，紧紧围绕主题主线，以提高经济增长质量和效益为中心，稳中求进，开拓创新，按照市第十一次党代会提出的目标任务，始终以世界眼光谋划未来，以国际标准提升工作，以本土优势彰显特色，全面推进改革开放，加快经济结构调整，实施蓝色引领、全域统筹、创新驱动战略，推进文化强市和生态文明建设，着力保障和改善民生，促进经济持续健康发展，保持社会和谐稳定，加快建设宜居幸福的现代化国际城市。

　　主要预期目标是：全市生产总值增长10.5%，城乡居民收入增长11%，公共财政预算收入增长15%，固定资产投资增长20%，外贸进出口总额增长8%，社会消费品零售总额增长15%，城镇新增就业15万人，农村劳动力转移就业10万人，城镇登记失业率控制在4%以内，居民消费价格涨幅控制在3.5%左右，人口自然增长率控制在4‰以内，完成国家和省下达的节能减排目标任务。

　　着力做好以下八个方面的工作：

　　（一）加快实施蓝色引领战略，推动产业结构调整取得新进展

　　突出蓝色经济引领作用，以加快功能区建设、优化产业结构、提高产业整体素质为重点，以总投资5385亿元的204个重点项目为抓手，加快构建以蓝色高端新兴为特色的现代产业体系。

　　加快“一谷两区”建设。深化西海岸经济新区建设规划，抓好董家口70平方公里港区泊位、物流园区和疏港铁路等基础设施建设，新开工一批百亿级重大项目建设。争创董家口国家循环经济示范区，开工建设西海岸30平方公里中央商务区，加快中德生态园、中日韩创新产业园和保税港区配套区建设。开工建设30平方公里蓝色硅谷核心启动区，加快山东大学青岛校区、青岛海洋科学与技术国家实验室、国家深海基地、国家海洋设备质检中心等18个重点项目建设进度，创建国家各类海洋科技创新试点示范园区。开工建设红岛经济区红岛街道30平方公里发展片区，加快30平方公里软件科技城基础设施和大项目建设，加快建设科技人文生态新城。

　　大力发展蓝色产业。加快发展海洋生物、海洋新材料两大战略性新兴产业，发展壮大海洋设备、海洋船舶、海洋化工、海洋运输、海洋渔业、海洋旅游、海洋信息、涉海服务等优势产业，抓好蓝色生物医药产业园等八个海洋特色园区建设，推动140个重点项目建设，增强海洋资源开发利用能力，建设国际海洋强市。

　　促进高端制造业集约发展。提升发展轨道交通装备、船舶海工、石化化工等优势产业链，加快发展乘用汽车、卫星导航应用、民用航空、品牌游艇、远洋渔船、炼化一体等高端制造业，培育发展页岩油气装备等新兴制造业，着力发展集成装备制造业。抓好300个工业转型升级重点项目，培育20个百亿级先进制造业园区，引进高端产品和关键配套制造项目，壮大十条千亿级产业链。加快老城区工业退出，开工建设青钢迁建项目，推动青岛石化、海晶化工等企业迁建。抓好两化融合示范项目，加快信息网络技术应用的基础设施建设，推进三网融合和信息提速，统筹推进“智慧青岛”建设，促进信息化与工业化、城镇化、农业现代化的广泛融合。

　　扩大高端服务业总量。推进高端服务业十个千万平米工程建设，重点建设即墨商贸物流区等六大服务业功能区。以财富管理为特色建设区域性金融中心，加快建设金家岭金融新区和鳌山卫财富管理园区。优化金融生态环境，注重防范金融风险。开工建设西海岸国际航运中心、董家口国际水产品交易中心和冷链物流基地。提升科技服务业水平，加快清华科技园、光谷软件产业园、国际创新园、工业设计产业园建设。加快主城区中央商务区发展，建设总部经济聚集区和高端商贸区。完善旅游管理体制，加强崂山风景名胜区管理，加快邮轮母港城建设，新策划发展一批旅游大项目，提高旅游品牌美誉度，建设国际性旅游目的地城市。规划建设国际海洋会议中心和西海岸、红岛会展中心，着力打造海洋、亚太主题品牌展会，加快建设国际海洋会展名城。

　　培育发展战略性新兴产业。制定海洋生物医药、海水淡化装备、海洋新能源等产业发展规划，在海洋可再生能源利用等方面走在全国前面。加快物联网、下一代互联网、信息安全、新型显示和3D制造、机器人智能装备等产业化示范工程。实施新兴产业创投计划，推进新能源、新材料、新医药、新信息领域150个投资过亿元的新兴产业重点项目，提升新兴产业的规模和竞争力。

　　推进农业现代化。以创建国家级现代农业示范区为目标，以高端高质高效为引领，实施现代农业园区建设、农业产业化促进、农产品质量品牌提升、生态循环农业示范、智慧农业建设、农业机械化促进、农产品市场开拓、远洋渔业培育、农田节水灌溉、现代林业生态建设十大现代农业工程。创新农业经营体制机制，培育新型经营主体，发展多种形式的规模经营，抓好社区产业园发展。发挥好农业生产专业合作组织作用。以更有效的措施加强农产品生产过程的安全监管。高度重视粮食生产和流通，保障粮食安全。完善基层农技推广、信息化服务和基层水利服务体系。抓好大沽河沿岸现代农业产业带建设，完成好全国水利现代化城市试点工作。鼓励发展远洋捕捞，加快渔港建设，提升现代海洋渔业发展水平。

　　（二）加快实施全域统筹战略，推进城乡一体化发展再上新水平

　　坚持新型工业化、信息化、城镇化和农业现代化同步发展，坚持全域统筹发展，坚持推进均衡发展，把市北、李沧和平度、莱西作为统筹的重点区域，把全市老城区危旧房改造和新型农村社区建设及产业发展作为统筹的主要抓手，推进基本保障和公共服务均等化，加快缩小城乡差距、南北差距，努力让城乡居民共享宜居幸福的小康社会美好生活。

　　加快三城联动发展。做优做美东岸城区，加快推进中山路、啤酒文化休闲商务区、欢乐滨海城、交通商务区、世园生态都市新区等重点片区建设。做大做强西岸城区，推进开发区扩展区、保税功能拓展区、国际经济合作区等重点功能区建设。做高做新北岸城区，完成会展中心、文化体育中心和医疗健康中心的开工建设前期工作。抓好地铁2、3号线建设，开工建设蓝色硅谷城际铁路，完成西海岸城际铁路建设前期工作。按计划推进青荣铁路、青龙高速、新疆路快速路建设进度，完成铁路青岛北客站、海青铁路建设，重庆路改造段实现主线通车，力争开工建设青连铁路。完成胶东国际机场开工前期工作，规划建设临空经济区。推进全域公交统筹发展。开工建设环胶州湾沿岸绿道慢行系统，完善三城互联交通体系。编制主城区地下空间资源利用规划。主城区危旧房改造1.7万户，开工建设保障性住房1.5万套。开工建设主城区电网重点项目、即东天然气管线，推进华电三期、大唐天然气热电联产项目。继续开展“大干300天市容环境整治行动”，努力实现城市精细化管理，提升城市品质。

　　加快城乡统筹发展。实施城乡统筹五年规划方案，加快推进十个省级示范镇建设，推动区划调整后的镇驻地向小城市发展。坚持住房建设和产业园区、公共服务同步推进，新开工100个新型农村社区，新建农村住房2.5万套，完成农村危房改造3813户,规划和扶持建设1000个农村社区产业园。落实好强农惠农富农政策。抓好150个经济薄弱村为重点的农村扶贫开发工作。做好新生代农民工的职业技能培训工作，多渠道增加农民收入。加快展开大沽河沿线和滨海蓝色经济发展带、烟威青综合发展带、济潍青综合发展带建设，沿轴带形成一批各具特色的城镇组团和产业园区，推动全域统筹发展和县域经济整体提升。

　　（三）加快推进改革开放，激发经济社会发展新活力

　　全面推进改革。处理好政府和市场的关系，破除影响科学发展的思维定势、利益格局和体制机制，增强改革的系统性、整体性、协同性。加强各类国有资产的监管，调整国有资本布局。国有资本从一般性竞争领域加快退出，投向国计民生重要行业和关键领域。推进股权多元化，改革重组部分国有企业。改善国有企业监管，提高企业自我管理、有效决策、应对竞争的能力。对小微企业特别是科技型小微企业继续给予扶持，落实到位鼓励民间投资各项政策，让各种所有制经济依法平等使用生产要素、公平参与市场竞争并受到法律保护。发展创投基金，鼓励企业上市，活跃金融市场，扩大社会融资总规模。创新重点发展区域、经济园区以及重大项目建设投融资机制，形成开发与金融良性互动。青岛经济技术开发区省级综合配套改革试点和国家级开发区体制机制创新试点工作要取得实效。加快农村集体经济组织产权制度改革，积极推进农村土地承包权、建设用地使用权、宅基地使用权、集体林权确权和流转制度改革，建立农村产权交易平台，促进金融资源与农村资源融汇，助力新农村建设。推进行政体制自我改革，减少市级行政审批，经济管理权限该下放、能下放的要下放，行政审批事项该取消、能取消的要取消。开展各功能发展区和镇级扩权工作。纵深推进城市建设、市政公用、科教文卫等领域的改革，加快事业单位分类改革步伐。加快户籍制度改革，完善流动人口居住证制度，有序推进农业转移和常住人口市民化。

　　坚定不移、更大力度地扩大开放。整合招商资源，创新招商方式，完善招商促进体系，继续引进世界500强、国内行业百强企业。引资、引技、引智相结合，吸引投资基金，利用海外融资，扩大引进外资规模。探讨境外设立青岛工商服务中心，建立双向投资贸易合作与服务新模式，推动“走出去”，支持企业开发境外资源、国际研发合作、跨国并购投资和海外工程承包。稳定出口与扩大进口并重，加快传统贸易转型，大力发展国际转口贸易和服务贸易，创建国际贸易中心试点城市。开发新兴市场，推进出口基地建设，继续扶持外贸企业扩大出口。推进省级开发区进位升级，办好和争取新设立各类国家级经济技术开发区。推动海关特殊监管区整合、扩大和提升发展，加快建成汽车整车进口口岸。推进口岸大通关工程，强化关贸、检贸、税贸和银贸合作机制。加快建设中日韩地方经济合作示范城市，深化国际友城交流和区域城市战略合作。做好对口支援工作。

　　（四）加快实施创新驱动发展战略，增强内生增长的新动力

　　加快创新平台建设。抓好千万平米孵化器建设，新开工170万平方米、竣工120万平方米。加快引进中科院长春应化所、天时海洋工程与装备研究院等国内外高端研发机构，大力引进船舶、航天航空等央企军工科技资源。推进国家海洋技术交易服务与推广中心、中船重工725所等重点创新平台建设，加快软件、橡胶新材料、海洋医药等20个市级公共研发平台建设。做好国家技术创新工程试点工作，完善以企业为主体、市场为导向、产学研相结合的技术创新体系。推进国家工程技术研发中心、企业国家重点实验室等研发机构建设，培育60家企业研发中心和50家企业技术中心，构建5个产业技术创新战略联盟，实施1000项技术创新重点项目，推进70项关键技术攻关。尊重、爱护、支持优秀企业家，鼓励企业家发挥好在科技创新中的领军作用。实施标准化战略和质量强市战略。推进国家知识产权示范城市建设，强化科技和金融结合，切实提高科技成果转化能力。

　　加快人才高地建设。抓实百万人才引进行动计划和青岛英才211引才工程，全年力争引进千名博士、万名硕士、十万名本科生。办好蓝色经济国际高端人才项目洽谈会。扩大金蓝领培育计划规模。加快建设海洋人才市场。完善人才政策，创新人才培养开发、发现引进、评价选拔、激励保障机制。加快人才公寓建设，完善和落实好高端人才住房政策，营造创新创业良好环境，依靠精英荟萃、人才聚集，加快建设创新型城市。

　　（五）加快文化产业和文化事业发展，推动文化强市建设迈上新台阶

　　推动普及社会主义核心价值观。用社会主义核心价值体系引领社会思潮、凝聚社会共识。弘扬民族精神和时代精神，开展好爱国主义、集体主义、社会主义教育，倡导富强、民主、文明、和谐，倡导自由、平等、公正、法治，倡导爱国、敬业、诚信、友善。加强公民思想道德建设，弘扬“微尘”、“红飘带”精神，培育知荣辱、讲正气、做奉献、促和谐的良好风尚。鼓励开展群众性精神文明创建活动，做好第四轮全国文明城市创建工作。抓好未成年人思想道德建设。加强政务诚信、商务诚信、社会诚信和司法公信建设。加强科普工作，提高居民科学素养。

　　推动文化大发展大繁荣。实施文化产业、公共文化服务和文化精品创作、文化人才培养引进“三大跨越工程”。着力创建国家公共文化服务体系示范区，加快市文化中心等公共文化重点设施建设，完善市、区、镇街、社区四级公共文化服务网络，推动城乡公共文化服务设施向社会免费开放。组织实施好文化惠民工程，举办群众文化艺术节、“三下乡”等系列文化活动。加强城市风貌和文化遗产保护，编制青岛历史建筑群和即墨故城遗址保护规划，加快国家水下文化遗产保护青岛基地建设，建设青岛山炮台一战遗址公园。推进千万平米文化产业园建设，培育国际动漫产业园、建筑创意产业园等十个重点文化创意产业园区，引进高端文化创意产业项目，力争国家级数字出版基地落户。鼓励各类企业进入文化产业领域。抓好国家文化和科技融合示范基地建设，促进文化与科技、文化与旅游融合发展，培育和壮大新兴文化业态。加大对外文化交流。加强文化市场管理，深入开展扫黄打非。促进网络文化健康发展。建设文化环保城市。筹办好第十届中国艺术节。

　　（六）加快生态文明建设，促进美丽青岛建设取得新成效

　　树立尊重自然、顺应自然、保护自然的生态文明理念，打造美丽中国标杆城市，使青岛展现出人与自然的和谐之美、城市建设的雅致之美、城乡一体的宏大之美、宜居幸福的愉悦之美。

　　促进资源节约和环境保护。统筹布局，加快形成节约资源和保护环境的空间格局、产业结构、生产方式、生活方式。严格保护耕地，推进土地集约化利用。强化污染排放总量控制，完善环境保护机制。实施低碳发展规划，加快淘汰“两高一资”落后产能。推进万家企业节能低碳行动和重点节能工程，推进建筑节能和可再生能源建筑应用示范。抓好全国低碳交通运输体系试点城市建设。加快循环经济示范工程和国家“城市矿产”示范基地建设。推进生态市、镇、村创建工作，建设镇村污水处理设施，实施垃圾统一集中收处。科学用药用肥，减少农业面源污染。开展生活垃圾分类收集试点，推进餐厨垃圾和建筑废弃物资源化处理。强化水、大气、噪声等污染防治，控制细颗粒物等主要污染物浓度，保持饮用水水源地水质优良。

　　推进生态建设。加快形成生态间隔标准制度体系和生态投入长效机制，打造城区之间、组团之间、产业功能区之间的生态间隔带。高质量完成世园会园区建设，统筹做好各项试运营管理工作，创建国家生态旅游示范区。创建国家森林城市，继续实施城乡植树增绿，提升全域森林生态体系建设水平。抓好骨干道路和水系生态绿化，加快万亩林场建设，干好主城区绿化提升工程。完成大沽河治理防洪主体工程，搞好造林绿化和湿地修复，加快形成大沽河生态中轴。制定实施环湾保护三年行动计划，加强重点海湾、海岸带、海域、湿地、海岛环境整治与生态修复，开工李村河、楼山河、墨水河等河道综合整治工程，严格控制入海排污总量，切实保护海洋生态环境。把资源、环境、生态指标纳入经济社会发展评价体系，健全责任追究和环境损害赔偿制度，努力提高人民群众对生态环境的满意度。

　　（七）加快改善民生和创新管理，推动社会建设取得新进步

　　坚持保基本保公共保普惠保均等，围绕实现学有优教、劳有应得、病有良医、老有颐养、住有宜居的目标，着力解决人民群众最迫切、最期盼、最忧心的事情，努力让发展成果更多更公平地惠及群众。今年计划安排民生投入165.6亿元。办好12件市办实事。

　　坚持教育优先发展。继续加大教育投入，建立生均公用经费稳定增长和教师交流的长效机制。加大学前教育投入，新建、改扩建幼儿园200所，免费为幼儿园生活环境进行检测，提高普惠性民办幼儿园财政补助标准。新增农村专用校车600辆。加快现代化学校建设，力争30%的普通中小学达到市现代化学校建设标准。开工建设青岛外语学校、青岛美术学校等中学新校区。全面完成中小学校舍安全工程。继续推进职业教育改革试点，建设10个高职特色专业群，培育现代制造业等3个示范性职教集团。积极支持民办教育。加强与高校的合作共建，推进在青普通高校重点学科市校共建工作。积极引进国内外优质教育资源。支持农村教育和特殊教育发展，推动新市民子女平等接受教育。全面推进素质教育，加强教师队伍建设，提高学生身心健康水平。

　　增加就业和改善居民生活。引导企业建立职工工资收入正常增长机制，调整企业退休人员基本养老金，适时提高城乡低保标准。完善企业职工和城乡居民基本养老保险制度，完善失地农民社会养老保险。推进城乡就业信息和人力资源市场一体化，重点解决好高校毕业生、农村转移劳动力、城镇就业困难人员和退役军人就业，实现更高质量的就业，新增城乡就业25万人。突出以创业带动就业，开工建设市创业就业实训基地，建设创业大学和博士创业园。促进居民消费便利化，降低消费成本，提高消费质量。实施城乡商贸流通网络体系、消费服务体系和鲜活农产品流通体系提升计划，规划建设区域性农产品交易中心，市区新建改造15处农贸市场。开展城市购物节、满意消费惠万家、农贸市场整治年等活动。继续按计划保质保量抓好各类保障房建设。市区新增供热面积560万平方米，建立农村低保家庭冬季取暖补助制度。新建农村无害化卫生厕所1万座。新增更新节能环保型公交车400辆。建设公共停车场9座、公共停车位2488个，推进交通智能化管理。

　　提高城乡居民健康水平。推进8所县级公立医院综合改革试点，全面取消以药补医。巩固完善基本药物制度。落实长期医疗护理保险制度，建立城镇居民大额医疗补助制度，做好农村大病医疗救助工作。提高城镇居民医保和新农合最低筹资标准，提高政策范围内住院费用支付比例。提高公共卫生服务均等化水平，增加基本公共卫生项目补助资金。健全基层医疗卫生服务体系，实施公立医院标准化体系建设，加大民营医院扶持力度。推进百万平米医疗设施建设计划，抓好山东大学齐鲁医院青岛院区规划建设，推进市第三人民医院、市妇儿医院二期和市立医院东院二期等重点项目建设。实施医疗技术、医疗服务双提升工程。建设防治结合型医疗卫生机构，重视中医药事业发展，开展全民健康促进行动，倡导健康的生活方式。

　　促进各项事业全面发展。加强应对人口老龄化战略研究，积极探索多元化养老模式，推进养老基础设施建设，新增床位5200张以上，加快市儿童福利院、市优抚医院、市社会福利院整体改造。规划建设残疾人综合服务中心，实施困难残疾人家庭无障碍设施改造，为成年重度残疾人发放就业生活补贴。完善社会救助体系，支持发展慈善事业。新建社区健身场所全覆盖，促进开展群众性体育健身活动。做好全运会参赛工作。做好第二届世界休闲体育大会筹备工作。统筹做好人口与计划生育工作，免费实施全市孕产妇产前筛查和新生儿疾病筛查，提高出生人口素质，促进人口均衡发展。促进妇女儿童事业健康发展。搞好第三次全国经济普查。开展好“六五”普法工作。积极做好新形势下的双拥工作，扎实推进国防教育和民兵预备役建设，实现军地融合发展。依法管理宗教事务，促进民族团结进步。做好外事、侨务、对台、统计、档案、史志、仲裁、人防、气象、防震减灾等工作，为推进科学发展服务。

　　加强和创新社会管理。深入推进平安青岛建设，健全完善立体化治安防控体系，推进社会治安“天网”工程建设。严格履行安全生产责任制，开展好“问安青岛”全民参与安全监督活动。改革完善食品药品安全监管体制机制，更大力度地开展“食安青岛”整治行动。依法管理好信息网络。进一步畅通信访渠道，完善矛盾纠纷化解机制。健全各类突发事件应急体系。社会管理重心下移，构建以城乡社区为重点的基层社会管理服务体系，为社区居民提供入托、入学、就业、养老、助残、婚恋等便民服务。改善对流动人口和特殊人群的管理服务。支持发展好志愿者队伍，鼓励深化和扩大志愿服务。引导好各类协会、商会等社会公益组织健康有序发展。

　　（八）加快提高政府自身建设水平，树立为民务实清廉新形象

　　积极寻标对标达标夺标创标，持续加速提升创新增效落实，努力建设人民满意的服务型政府。

　　推进依法行政。健全科学、民主、依法决策机制和程序，完善决策问责和纠错制度。大力推进政务公开，让行政权力始终在阳光下运行。继续开展向市民报告、听市民意见、请市民评议活动，充分发挥网络在线问政、行风在线、民生在线、政务服务热线等互动平台作用，及时回应群众诉求。改进和完善政府决策，扩大公众参与度。规范行政执法行为，减少自由裁量并规范公开，完善执法评议考核机制，实现行政复议权相对集中。各级领导干部都要始终坚持有法必依、执法必严、违法必究，绝不允许以言代法、以权压法、徇私枉法。

　　提高服务效能。深入开展庸懒散慢拖瞒专项治理，按照“六个力倡、六个反对”的要求，不断优化政治经济生态。坚持好市政府联系群众、联系企业制度，完善电子政务公共服务平台，提升服务标准，优化服务流程，强化服务评价，增强政府服务市民、服务企业和服务项目的能力。创新行政管理方式，强化绩效管理，提高服务效率。

　　加强作风建设。认真落实中央政治局关于改进工作作风、密切联系群众八项规定。按照中央、省委的统一部署，在市委的领导下，扎实开展以为民务实清廉为主要内容的党的群众路线教育实践活动，着力解决人民群众反映强烈的突出问题。大兴调查研究之风，领导干部要亲力亲为，深入到困难多、群众意见大的地方去研究问题、解决困难、指导工作。进一步改进会风文风，大力精简会议和文件。努力做到讲实话、干实事，善作为、勇担当，言必信、行必果，增强政府的公信力和执行力。

　　加强廉政建设。坚持标本兼治、综合治理、惩防并举、注重预防方针，推进惩治和预防腐败体系建设，深化科技防腐工作，做到干部清正、政府清廉、政治清明。强化对重点领域行政权力运行的监督，推进“三公”经费、行政经费公开工作，厉行节约，反对浪费，严格执行住房、车辆配备等规定。加强对资金、国有资产、公共资源交易等重要领域的监管，对财政直接投资的公用项目建设实行相对集中管理。推进对工程建设领域的治理、审计和监督检查。加大领导干部经济责任审计力度。查处大案要案，力求遏制腐败，始终保持清正廉洁。

　　政府的权力来自人民，必须对人民负责，受人民监督。要认真执行市人大及其常委会的决议、决定，坚持重大事项向人大报告、向政协通报制度，主动接受人大法律监督、工作监督和政协民主监督，切实做好人大代表建议、意见及政协提案办理工作。认真听取各民主党派、工商联和无党派人士意见。主动接受舆论监督和公众监督，有错必纠，有过必改，不断改进工作，提高行政质量。政府工作人员要始终坚持全心全意为人民服务的行动准则，始终保持奋发有为的精神状态，尽职尽责，努力工作，勇于奉献，不断创造让群众满意的新业绩。

　　各位代表！新的一年，新的愿景。让我们紧密团结在以习近平同志为总书记的党中央周围，在中共青岛市委的坚强领导下，以逢山开路、遇河架桥的精神，做好今年各项工作，为率先全面建成更高水平的小康社会，为加快建设宜居幸福的现代化国际城市而努力奋斗！
